# Effects of cytochalasin congeners, microtubule-directed agents, and doxorubicin alone or in combination against human ovarian carcinoma cell lines in vitro

**DOI:** 10.1186/s12885-015-1619-9

**Published:** 2015-09-10

**Authors:** Matthew Trendowski, Timothy D. Christen, Christopher Acquafondata, Thomas P. Fondy

**Affiliations:** Department of Biology, Syracuse University, 107 College Place, Syracuse, NY 13244 USA

## Abstract

**Background:**

Although the actin cytoskeleton is vital for carcinogenesis and subsequent pathology, no microfilament-directed agent has been approved for cancer chemotherapy. One of the most studied classes of microfilament-directed agents has been the cytochalasins, mycotoxins known to disrupt the formation of actin polymers. In the present study, we sought to determine the effects of cytochalasin congeners toward human drug sensitive and multidrug resistant cell lines.

**Methods:**

SKOV3 human ovarian carcinoma and several multidrug resistant derivatives were tested for sensitivity against a panel of nine cytochalasin congeners, as well as three clinically approved chemotherapeutic agents (doxorubicin, paclitaxel, and vinblastine). In addition, verapamil, a calcium ion channel blocker known to reverse P-glycoprotein (P-gp) mediated drug resistance, was used in combination with multiple cytochalasin congeners to determine whether drug sensitivity could be increased.

**Results:**

While multidrug resistant SKVLB1 had increased drug tolerance (was more resistant) to most cytochalasin congeners in comparison to drug sensitive SKOV3, the level of resistance was 10 to 1000-fold less for the cytochalasins than for any of the clinically approved agents. While cytochalasins did not appear to alter the expression of ATP binding cassette (ABC) transporters, several cytochalasins appeared to inhibit the activity of ABC transporter-mediated efflux of rhodamine 123 (Rh123), suggesting that these congeners do have affinity for drug efflux pumps. Cytochalasins also appeared to significantly decrease the F/G-actin ratio in both drug sensitive and drug resistant cells, indicative of marked microfilament inhibition. The cytotoxicity of most cytochalasin congeners could be increased with the addition of verapamil, and the drug sensitivity of resistant SKVLB1 to the clinically approved antineoplastic agents could be increased with the addition of cytochalasins. As assessed by isobolographic analysis and Chou-Talalay statistics, cytochalasin B and 21,22-dihydrocytochalasin B (DiHCB) demonstrated notable synergy with doxorubicin and paclitaxel, warranting further investigation in a tumor-bearing mammalian model.

**Conclusion:**

Cytochalasins appear to inhibit the activity of P-gp and potentially other ABC transporters, and may have novel activity against multidrug resistant neoplastic cells that overexpress drug efflux proteins.

## Background

**Fig. 1:**
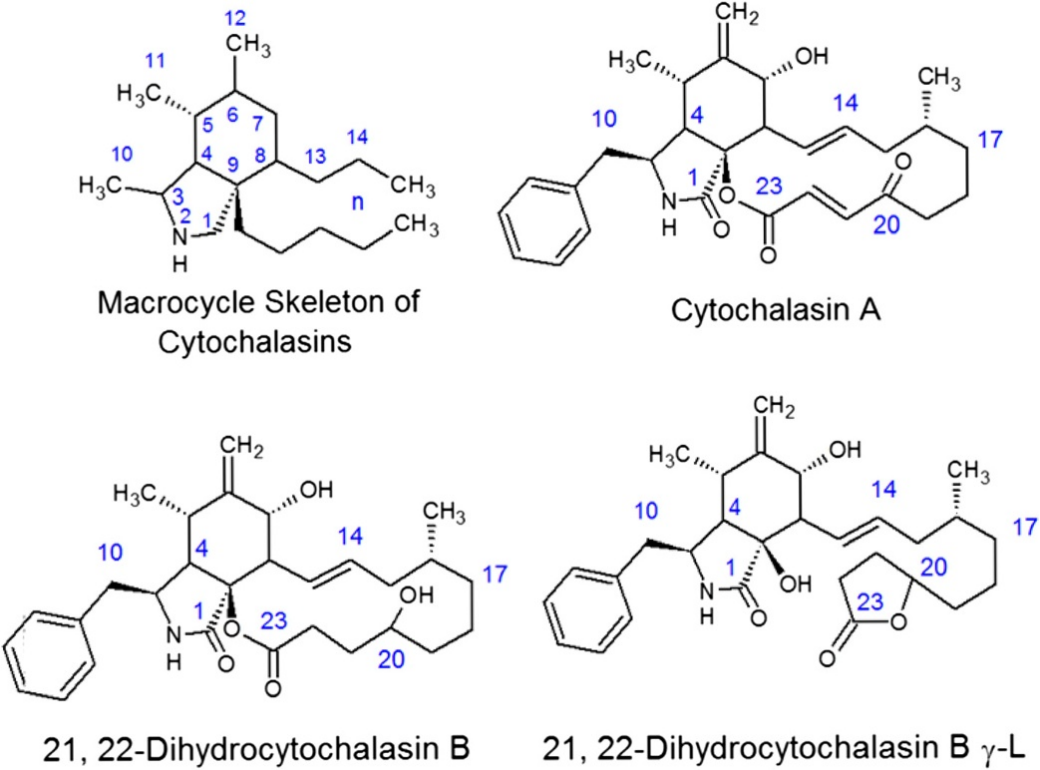
Molecular structure of the cytochalasin macrocycle and several congeners. The macrocycle skeleton of cytochalasins is provided to indicate the numbering system used for these congeners. In addition, the structure of 21,22-dihydrocytochalasin B is shown to indicate the differences in structure it has with the γ-lactone derivative. The α, β-unsaturated ketones of cytochalasin A runs from C-20 to C-23

Cytochalasins are mycotoxins known to disrupt the formation of filamentous (F)-actin, thereby preventing the formation of functional microfilaments. These congeners are characterized by a highly substituted perhydro-isoindolone structure that is typically attached to a macrocyclic ring [1]. More than 60 different cytochalasins from several species of fungi have been classified into various subgroups based on the size of the macrocyclic ring and the substituent of the perhydroisoindolyl-1-one residue at the C-3 position [2]; structures of representative cytochalasins are shown in Fig. 1. While most of our previous work has focused on cytochalasin B, there are many other congeners with similar activity toward microfilaments. As microfilament-disrupting agents, cytochalasins alter cell motility, adherence, secretion, drug efflux, deformability, morphology, and size, among many other cell properties critical to neoplastic cell pathology [1, 2]. In addition, two of the congeners (cytochalasins B and D) have shown partial specificity against neoplastic cells [3–10], consistent with the substantial differences known to exist between the microfilament biochemistry of neoplastic and normal cells [11, 12]. These differences in microfilament structure may be related to key neoplastic characteristics, including altered adherence, anchorage independent growth, invasiveness, and altered plasma membrane cytoskeletal interactions involving expression of oncoproteins [12, 13].

Previously, we have demonstrated that cytochalasin B and its reduced congener 21,22-dihydrocytochalasin B (DiHCB) are able to sensitize multidrug resistant P388/ADR murine leukemia cells to doxorubicin, with both congeners showing considerable drug synergy with the nucleic acid-directed agent [14]. In addition, prior research has indicated that cytochalasin B efflux is less affected by overexpression of ATP binding cassette (ABC) transporters than other cytotoxic drug classes (vinca alkaloids and anthracyclines) [15] that often exhibit drug resistance in the clinical setting. Based on these observations, it appears that cytochalasin B and potentially other cytochalasin congeners might be active against multidrug resistant neoplastic cells and might also be able to overcome resistance to other cytotoxic agents currently used in the clinical setting. Therefore, this study seeks to determine the effects of cytochalasin congeners toward drug sensitive and multidrug resistant human cancer cell lines.

SKOV3 is a human ovarian carcinoma cell line frequently used *in vitro* and also *in vivo* as a xenograft in immunosuppressed mice. SKOV3 cells have slight, but noticeable resistance to tumor necrosis factor, as well as to cisplatin and doxorubicin [16, 17]. The inherent drug resistance of SKOV3 cells can be dramatically elevated through progressively increasing exposure to either vinblastine or vincristine [18]. One of the most notable multidrug resistant cell lines, SKVLB1, is 2,000-fold more resistant to vinblastine, 10,000-fold more resistant to vincristine, 260-fold more resistant to doxorubicin, and 510-fold more resistant to the non-clinically approved colchicine when compared to the parental cell line [18]. Further, this increase in drug resistance is mirrored by increasing overexpression of P-glycoprotein (P-gp), a known ABC transporter [19, 20]. Since cytochalasin B efflux is notably resistant to P-gp overexpression, drug sensitive and drug resistant SK human ovarian carcinoma cell lines are ideal models to examine cytochalasin sensitivity in multidrug resistant cancers. These cell lines can also reveal potential drug synergism when cytochalasin congeners are combined with chemotherapeutic agents, or with calcium ion channel blockers known to inhibit P-gp drug efflux [20–24].

## Methods

### Preparation of human ovarian carcinoma cell lines

SK human ovarian carcinoma cell lines with varying levels of drug resistance were provided courtesy of Dr. Victor Ling (University of British Columbia, Canada). The level of drug resistance of the cell lines from lowest to highest is as follows: SKOV3 (parental cell line), SKVCR0.015, SKVCR0.1, and SKVLB1 (see Reference [18] for details on how drug resistance is acquired). All cell lines were seeded at 4 × 10^4^ cells/cm^2^ in 25 cm^2^ culture flasks, and suspended in 9 ml of RPMI 1640 complete medium containing 10 % newborn calf serum (GIBCO, Grand Island, NY, USA), 0.4 units/ml penicillin, 0.4 μl/ml streptomycin, and 250 μg/ml fungizone. Flasks were incubated in 5 % CO_2_ at 37 °C. During subculture, cells were trypsinized with 0.05 % trypsin-EDTA solution 1X (Sigma-Aldrich Corp., St. Louis, MO, USA) for 5 min at 37 °C, dislodged by a sharp knocking of the flasks during that period, washed, diluted to 10 ml with fresh complete medium, and 1 × 10^6^ cells were seeded into 25 cm^2^ culture flasks (4 × 10^4^ cells/cm^2^).

### Cytochalasin B preparation

Cytochalasin B was prepared from mold mattes of *Drechslera dematioidea* (ATCC® 24346) as previously described [13, 14, 25], and purified by preparative thin layer chromatography to greater than 99 % homogeneity after recrystallization from chloroform. Cytochalasin B and other cytochalasins prepared in our laboratory were characterized with ^1^H NMR spectroscopy (spectra not shown), and were compared to commercially acquired samples (Sigma-Aldrich Corp.) to ensure that the isolated products were of a suitable grade.

### 21, 22-Dihydrocytochalasin B Preparation

DiHCB was prepared by sodium borohydride reduction of cytochalasin B in methanol at 25 °C as previously described [14, 26]. The product was recovered as a chloroform-soluble fraction and crystallized from benzene:hexane. DiHCB was compared to a commercially purchased sample of DiHCB (Sigma-Aldrich Corp.) and cytochalasin B (Sigma-Aldrich Corp.) using reverse phase thin layer chromatography.

### Cytochalasin D preparation

Cytochalasin D was prepared from mold mattes of *Zygosporium masonii* (ATCC® MYA­3308) as previously described [14, 26], and purified by preparative thin layer chromatography to greater than 99 % homogeneity after recrystallization from chloroform.

### Cytochalasin C preparation

Cytochalasin C was prepared through an isomerization reaction of cytochalasin D using a Pd/charcoal catalyst at 25 °C as previously described [26]. After filtration of the charcoal catalyst, cytochalasin C was isolated from any remaining cytochalasin D in the reaction product using C-18 reverse phase thin layer chromatography plates with methanol:water, 75:25 *v*/*v* as mobile phase, followed by fluorescence quenching. A small amount of commercial cytochalasin C (Sigma-Aldrich Corp.) was characterized by reverse phase thin layer chromatography and recrystallized from acetone:hexane for comparison with the purified product.

### Preparation of other cytochalasin congeners

All other cytochalasin congeners (cytochalasins A, E, H, J, and DiHCBγ-L), were acquired commercially (Sigma-Aldrich Corp.). All cytochalasins used in the study were solubilized by dissolving 1.0 mg cytochalasin in 100 μl EtOH in conical 1.5 ml plastic centrifuge tubes. Once the entire compound went into solution, 100 μl of the 10 mg/ml cytochalasin/EtOH solution was diluted into 4.9 ml medium to give a 400 μM stock solution with 2 % EtOH.

### Preparation of clinically approved chemotherapeutic agents

Doxorubicin, paclitaxel, and vinblastine were acquired commercially (Sigma-Aldrich Corp.). Doxorubicin was solubilized in isotonic saline, paclitaxel in 1:1 100 % EtOH: Kolliphor EL, and vinblastine in sterilized water.

### Plate assay procedures

SK human ovarian carcinoma cells were tested for drug sensitivity using 24-well assay plates (Corning Life Sciences, Corning, NY, USA). Each well contained ~ 1000 cells in 1 ml medium. Agents were then dissolved in the wells at varying concentrations for 23 wells, while the last well remained untreated. The plates were incubated at 37 °C for the length of drug exposure, and then stained with methylene blue. This method was then used to assess varying inhibitory concentrations of each agent alone or in combination with another agent as described in [18]. The efficacy of the method was also confirmed with a XTT Cell Proliferation Assay Kit (ATCC® 30-1011 K).100 μl of cells were seeded per well into a flat-bottom 96-well microtiter plate in triplicate for each cell dilution. The plate was incubated for 24 h prior to addition of XTT solution. Cells were then incubated for an additional 2 h before the wavelength was read.

### Examining the effects of cytochalasins on microfilaments in neoplastic cells

Cytochalasins were assessed for their ability to inhibit the formation of F-actin by examining the ratio of F-actin to monomeric globular (G)-actin found within the SK human ovarian cancer cell lines prior to and after treatment. The F-actin to G-actin ratio was determined with the G-Actin/F-Actin In Vivo Assay Biochem Kit (Cytoskeleton Inc., Denver, CO, USA). After being treated, cells were lysed with LAS2 buffer (1 ml lysis and F-actin stabilization buffer, 10 μl of the 100 mM ATP stock solution, and 10 μl of the 100× protease inhibitor cocktail stock solution) on ice for 10 min. Cells were collected and the cell extracts were centrifuged at 4 °C for 75 min at 16,000 g to separate the F-actin and G-actin pools. The supernatants of the extracts were collected and designated as the G-actin pool. The pellets were resuspended in ice-cold actin depolymerization buffer and designated as the F-actin pool. Equal amounts of both the supernatant (G-actin) and the resuspended pellet (F-actin) were subjected to Western blot analysis with the use of an anti-β-actin antibody.

### Assessing the inhibitory activity cytochalasins have toward ATP binding cassette transporters

To determine whether cytochalasins exert antineoplastic activity via inhibition of P-gp and other ABC transporters, reverse transcription polymerase chain reaction (RT-PCR) was used to quantify the RNA levels of three ABC transporters; P-gp (ABCB1), Multidrug resistance-associated protein 1 (MRP1; ABCC1) and multidrug resistance-associated protein 2 (MRP2; ABCC2). Total RNA was extracted from cells according to instructions provided in the RNeasy Mini kit (Qiagen Inc., Valencia, CA, USA). Successfully extracted RNA was then dissolved in diethylpyrocarbonate/water. Absorption values were read at 260 nm and 280 nm using a UV spectrophotometer. Acquired RNA was converted into cDNA according to the instructions provided in the RT-PCR kit (Life Technologies, Grand Island, NY, USA), and primers used for RT-PCR are shown in Table 1. The reaction was carried out under the following conditions: denaturation at 95 °C for 5 min with an additional 15 s at 94 °C, and a 30 s annealing at 60 °C. Targets genes were directly quantified to the reference gene β-actin, since cytochalasins do not significantly affect the RNA expression levels of actin [1].

**Table 1:** Primer sequences used for reverse transcription polymerase chain reaction to quantify RNA expression of ATP binding cassette transporters in SK human ovarian carcinoma cell lines

Gene and NCBI ID	Forward primer	Reverse primer	T_A_ (°C)
ABCB1 (5243)	5′-GCC CTT GGA ATT ATT TCT TT-3′	5′-TGG GTG AAG GAA AAT GTA AT-3′	52
ABCC1 (4363)	5′-ATG TCA CGT GGA ATA CCA GC-3′	5′- GAA GAC TGA ACT CCC TTC CT-3′	51
ABCC2 (1244)	5′-GGA ACA ATT GTA GAG AAA GGA TC-3′	5′-CAC AAA CGC AAG GAT GAT GAA GAA-3′	55

In addition to examining the effects of cytochalasins on the RNA expression of ABC transporters in human ovarian carcinoma cells, direct inhibitory activity toward drug efflux pumps was assessed with rhodamine 123 (Rh123; 6-amino-9-(2- methoxycarbonylphenyl) xanthen-3-ylidene]azanium chloride). Cells were initially plated at 2 × 10^5^ cells/cm^2^ in 24-well plates and allowed to reach 80 % confluence. To assess the ability of cytochalasins to potentiate Rh123 accumulation, cells were incubated with 5 μM Rh123 in the presence and in the absence of cytochalasins or the known P-gp inhibitor verapamil for varying lengths of time. At each time point, cells were collected, washed, resuspended in ice-cold phosphate-buffered saline and kept on ice before fluorescence intensity was measured with flow cytometry. Cells were also assessed for their ability to efflux accumulated Rh123 in the presence of cytochalasins or verapamil by initially incubating the cells with 5 μM Rh123 for 30 min and then washing the cells twice with ice-cold PBS before being resuspended in fresh Rh123-free medium with and without agents. Cells were collected at various time points and were then washed and resuspended in ice-cold PBS before being analyzed by flow cytometry.

### Determining the extent of drug synergy between cytochalasins, doxorubicin, and paclitaxel

To assess whether cytochalasin B or DiHCB synergizes with doxorubicin or paclitaxel against SK human ovarian carcinomas *in vitro*, cells were treated alone, or in combination for 48 or 96 h. IC_50_ values were assessed by both the methylene blue and XTT assays. In addition, the Chou-Talalay method for assessing drug synergism was implemented to determine the combination index (CI), dose reduction index (DRI), and fraction affected (Fa). As indicated in [27], synergism was assessed with the following values: CI < 1 (synergy) CI = 1 (additive) CI > 1 (antagonism). In addition, DRI > 1 is representative of favorable dose reduction, while DRI < 1 is representative of unfavorable dose reduction [27].

## Results

### Comparison of cytochalasin congeners and other antineoplastic agents against SK human ovarian carcinoma cell lines

There were notable differences in cytotoxicity between cytochalasin congeners and other antineoplastic agents against the human ovarian carcinoma cell lines. Table 2 shows the comparative IC_90_ values and resistance indices for nine cytochalasin congeners and for doxorubicin, paclitaxel, and vinblastine. While doxorubicin, paclitaxel, and vinblastine all had comparatively low IC_90_ values against the parent SKOV3 cell line after a 96 h exposure indicative of relatively high efficacy, (11 nM, 2.8 nM, and 1.9 nM, respectively), these agents had relatively higher IC_90_ values against the intermediately drug resistant SKVCR0.015 and SKVCR0.1 lines (125 nM and 125 nM for doxorubicin, 8 nM and 4 nM for paclitaxel, and 10 nM and 20 nM for vinblastine, respectively), indicative of drug resistance. The SKVLB1 cell line showed even higher resistance to these three agents exhibiting much higher IC_90_ values (2900 nM, 3900 nM, and 3100 nM, respectively; Table 2). The profound differences in cytotoxicities of these agents against the drug sensitive parental SKOV3 line compared to the multidrug resistant SKVLB1 line produced very high resistance indices (RI values). These RI values ranged from 264 for doxorubicin to 1,400 and 1,600 for paclitaxel and vinblastine for SKVLB1 in comparison with SKOV3.

**Table 2:** Effects of clinically approved natural product antineoplastic agents and of cytochalasin congeners on drug-sensitive and multidrug-resistant human ovarian carcinoma lines

Cell line	SKOV3^a^	SKVCR0.015		SKVCR0.1		SKVLB1	
IC concentrations (nM) at 96 h	IC_90_	IC_90_	RI	IC_90_	RI	IC_90_	RI
Doxorubicin	11	125	11	125	11	2900	264
Paclitaxel	2.8	8	3	4	1.4	3900	1400
Vinblastine	1.9	10	5	20	10	3100	1600
Cytochalasin A	1000	1000	1	500	0.5	750	0.75
Cytochalasin B	2250	1500	0.67	1250	0.56	3000	1.33
21, 22-Dihydrocytochalasin B	3000	2400	0.8	1600	0.53	5000	2.5
21, 22-Dihydrocytochalasin B γ-L	40000	30000	0.75	30000	0.75	40000	1
Cytochalasin C	330	250	0.76	125	0.38	8000	24
Cytochalasin D	80	80	1	320	4	8000	100
Cytochalasin E	200	ND		ND		1600	8
Cytochalasin H	100	ND		ND		1600	16
Cytochalasin J	800	ND		ND		3200	4

*ND* not determined, *RI* resistance index compared to parental SKOV3 line

^a^RI = 1 for all agents for SKOV3 line

**Fig. 2:**
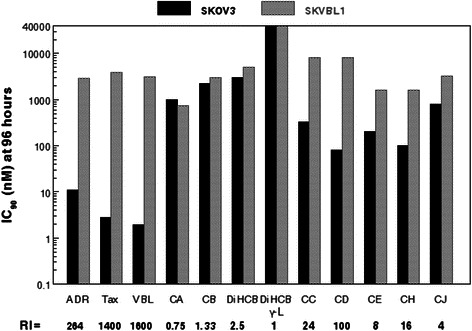
Comparison of clinically approved chemotherapeutic agents and cytochalasin congeners against SKOV3 drug sensitive and SKVLB1 multidrug resistant human ovarian carcinomas. Abbreviations used are as follows: ADR (Adriamycin; doxorubicin), Tax (paclitaxel), VBL (vinblastine), CA (cytochalasin A), CB (cytochalasin B), DiHCB (21,22-dihydrocytochalasin B), CC (cytochalasin C), CD (cytochalasin D), CE (cytochalasin E), CH (cytochalasin H), and CJ (cytochalasin J). The concentration of each agent in nM needed to produce an IC_90_ value at 96 h for either cell line is represented on a logarithmic scale. The resistance index (RI) of each compound against SKVLB1 is indicated in bold underneath the abbreviations

By contrast, cytochalasins did not show this marked increase in resistance against the multidrug resistant SKVLB1 line (Table 2). In fact, cytochalasin A produced a lower IC_90_ value against SKVLB1 (750 nM) than against SKOV3 (1,000 nM). Interestingly, the most cytotoxic congeners with IC_90_ values of 330 nM or less against drug sensitive SKOV3 (cytochalasins C, D, E, and H) had higher RIs against SKVLB1 than did cytochalasins A, B, J, DiHCB, or DiHCBγ-L; those with higher initial IC_90_ values (lower initial cytotoxicity against SKOV3). Nevertheless, the congener with the highest RI against SKVLB1, cytochalasin C (RI = 100 against SKVLB1), still had a considerably lower RI than the clinically approved chemotherapeutic agents (doxorubicin RI = 264, paclitaxel RI = 1,400, and vinblastine RI = 1,600). The differences in cytotoxicity expressed as IC_90_ values along with the respective RI values as shown in Table 2 are further highlighted in Fig. 2, which uses a logarithmic scale to compare the cytotoxicities of agents against SKOV3 and SKVLB1.

### Effects of a calcium ion channel blocker on cytochalasin-mediated cytotoxicity

As shown in Table 3, the calcium ion channel blocker verapamil increased the drug sensitivity of parental SKOV3 to cytochalasins A, B, and DiHCB by 1.2 to 2-fold. Sensitivity to cytochalasins C and D was not enhanced. With respect to the highly drug-resistant SKVLB1 line, verapamil did not notably increase cytochalasin A cytotoxicity. This presumably reflects the fact that cytochalasin A has slightly enhanced cytotoxicity for SKVLB1 than it does for SKOV3.

**Table 3:** Effects of verapamil on the sensitivity of human ovarian carcinoma cell lines to cytochalasin congeners

Cell line	SKOV3		SKVCR0.015		SKVCR0.1		SKVLB1	
IC concentrations (μM) at 96 h	IC_90_	FS	IC_90_	FS	IC_90_	FS	IC_90_	FS
Cytochalasin A								
Alone	0.8	-	0.8	-	0.2	-	0.75	-
15 μM Verapamil	0.4	2	0.4	2	0.1	2	ND	
30 μM Verapamil	0.4	2	0.4	2	0.1	2	0.75	-
Cytochalasin B								
Alone	2.3	-	1.5	-	1.1	-	3	-
15 μM Verapamil	1.2	1.9	1	1.5	0.55	2	ND	
30 μM Verapamil	1.1	2	0.75	2	0.55	2	2	1.5
21, 22-Dihydrocytochalasin B								
Alone	3	-	2.4	-	1.6	-	5	-
15 μM Verapamil	2.5	1.2	1.5	1.6	1.0	1.6	ND	
30 μM Verapamil	2	1.5	1.3	1.8	0.8	2	3.2	1.57
Cytochalasin C								
Alone	0.25	-	0.25	-	0.25	-	8	
15 μM Verapamil	ND		ND		ND		ND	
30 μM Verapamil	0.25	1	0.25	1	0.125	2	0.25	32
Cytochalasin D								
Alone	0.08	-	0.08	-	0.1	-	8	
15 μM Verapamil	0.08	1	0.06	1.5	0.1	1	ND	
30 μM Verapamil	0.08	1	0.04	2	0.075	1.5	0.13	64

*ND* not determined, *FS* fold sensitization

**Fig. 3:**
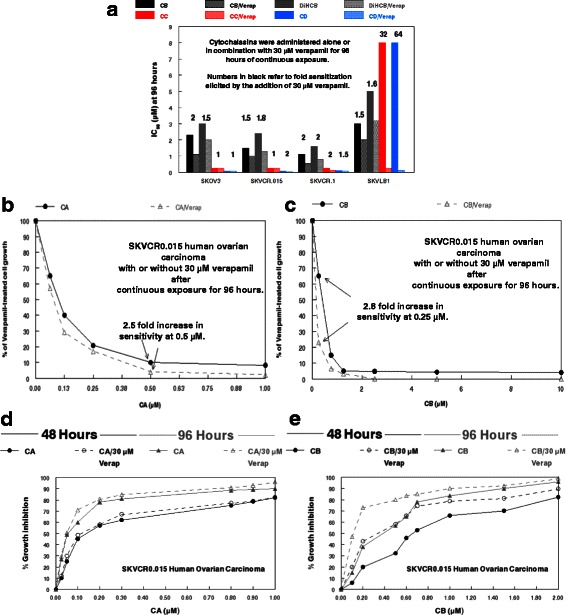
Effects of verapamil and cytochalasins on SK human ovarian carcinoma cell lines. **a** Effects of verapamil on potentiating SK human ovarian carcinoma sensitivity to cytochalasins B, C, D, and 21,22-dihydrocytochalasin B. Cell lines are arranged in order of increasing drug resistance from left to right. The IC_90_ concentrations are given in μM. Numbers in black refer to fold sensitization elicited by the addition of 30 μM verapamil. **b** Effects of cytochalasin A-alone or in combination with 30 μM verapamil against SKVCR0.015 cells. The values are taken as a percentage of 30 μM verapamil-treated cells. **c** Effects of cytochalasin B-alone or in combination with 30 μM verapamil against SKVCR0.015 cells. The values are taken as a percentage of 30 μM verapamil-treated cells. **d** Dose response of cytochalasin A-alone or in combination with 30 μM verapamil on the growth of SKVCR0.015 cells. **e** Dose response of cytochalasin B-alone or in combination with 30 μM verapamil on the growth of SKVCR0.015 cells. For panels **d** and **e**, agents were administered for either 48 or 96 h, as indicated in the graphs

Sensitivity of SKVLB1 to cytochalasin B or DiHCB was increased by 1.5 fold. Very strikingly, the sensitivity of SKVLB1 to cytochalasins C and D was markedly increased by 32- and 64-fold respectively. The SK lines with intermediate resistance to SKVLB1 (SKVCR0.015 and SKVCR0.1) showed a 1.5 to 2-fold increase in sensitivity to cytochalasins A, B, and DiHCB when administered in combination with 15 μM verapamil, and cytochalasin D had a similar increase in sensitivity with 30 μM verapamil. In addition, verapamil increased the sensitivity of SKVCR0.1 to cytochalasin C by 2-fold, but did not affect cytochalasin C with respect to SKVCR0.015. The effects of 30 μM verapamil on SK human ovarian carcinoma sensitivity to cytochalasins B, C, D and DiHCB are further highlighted in Fig. 3a. Verapamil enhanced the cytotoxicities of cytochalasin B and DiHCB in all four cell lines tested (black and grey bars), and it enhanced the cytotoxicities of cytochalasins C and D against SKVCR0.1 by 1.5 to 2-fold (red and blue bars). The dramatic increases in cytotoxicities of 32- to 64-fold noted above for cytochalasins C and D against the highly drug resistant SKVLB1 line when treated in combination with 30 μM verapamil are clearly apparent in Fig. 3a.

This increase in cytotoxicity potentiated by 30 μM verapamil in a 96 h exposure is exhibited at multiple concentrations of cytochalasin A against SKVCR0.015 (Fig. 3b), as well as with the less potent cytochalasin B against the same cell line (Fig. 3c). After continuous exposure for 96 h, 30 μM verapamil produces a 2.5-fold increase in cytotoxicity of cytochalasin A at 0.5 μM, and a 2.8-fold increase in cytotoxicity of cytochalasin B at 0.25 μM. The potential increase in cytotoxicity facilitated by verapamil is further highlighted in dose response growth curves of SKVCR0.015 cells after being treated alone with cytochalasins A or B, or concomitantly with 30 μM verapamil for 48 or 96 h. While the addition of verapamil does not considerably increase the percent growth inhibition at most time points for cytochalasin A (Fig. 3d), the calcium ion channel blocker does potentiate cytochalasin B mediated growth inhibition, particularly at lower concentrations (Fig. 3e).

### Effects of cytochalasins and verapamil on the F/G-actin ratio found in SK human ovarian carcinoma cells

**Fig. 4:**
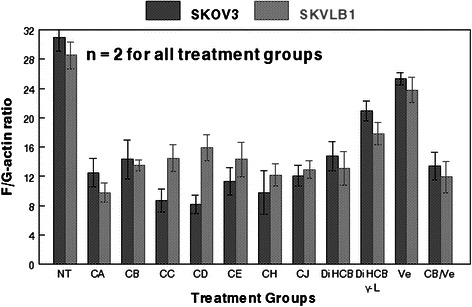
Effects of cytochalasin congeners on the F/G-actin ratio of SKOV3 and SKVLB1 human ovarian carcinomas. Abbreviations are the same as those used in Fig. 2, except for the addition of Ve (verapamil). All cytochalasins were administered at their IC_90_ value of 96 h continual exposure, and Ve was administered at 30 μM. The F/G-actin ratio of both SKOV3 and SKVLB1 cells was assessed 24 h post-administration. Bars represent standard error of the mean (SEM) for each treatment group

As expected, cytochalasins demonstrated varying levels of microfilament inhibition against the parental SKOV3 cells, with the more potent agents potentiating lower F/G-actin ratios (Fig. 4). In agreement with their IC_90_ values against SKOV3, cytochalasin D elicited the lowest F/G actin ratio after 24 h (8.2), while DiHCB γ-L elicited the highest (20.9). Interestingly, it appeared that 30 μM verapamil had a small, but notable effect on the F/G-actin ratio of SKOV3 cells. The efficacy of various cytochalasin congeners to inhibit actin polymerization was notably different in multidrug resistant SKVLB1. Although the cell line had a smaller, but notable baseline F/G-actin ratio than its parental counterpart (27.5 to 31), SKVLB1 was less sensitive to cytochalasin D inhibition (15.9), but much more sensitive to cytochalasin A (SKOV3: 12.5; SKVLB1: 9.8) and DiHCB γ-L (17.8), as was indicated by the four day IC_90_ concentrations. The F/G-actin ratio potentiated by cytochalasin B/verapamil was lower than cytochalasin B-alone for both SKOV3 (13.4 to 14.3) and SKVLB1 (11.9 to 13.5). The notable decrease in the F/G actin ratio elicited by the concomitant administration of cytochalasin B/verapamil follows the same pattern observed in the cytotoxicity assays of Fig. 3, suggesting verapamil may have a slight, but notable influence on cytochalasin B-mediated cytotoxicity.

### Assessment of ATP binding cassette transporter overexpression in SK human ovarian carcinomas and the inhibitory effects of cytochalasins and verapamil

**Fig. 5:**
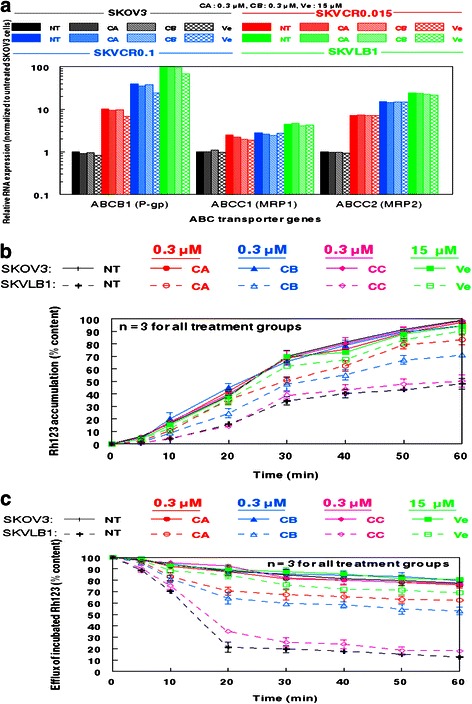
Effects of cytochalasin congeners and verapamil on RNA expression of ATP binding cassette proteins and the efflux of rhodamine 123 in SK human ovarian carcinoma cell lines. **a** Relative RNA expression of ABCB1 (P-gp), ABCC1 (MRP1), and ABCC2 (MRP2) in SK human ovarian carcinoma cell lines prior to and after treatment with cytochlalasins as assessed by RT-PCR. Primer sequences used in the reaction are shown in Table 1. **b** Cells were treated with the indicated agents, and then exposed to Rh123. **c** Cells were incubated with Rh123, and then placed in fresh medium. The concentrations of each agent used are indicated in the individual panels. Bars represent SEM for each treatment group

RT-PCR quantification of ABC transporters revealed that P-gp was substantially overexpressed in the drug resistant derivatives of SKOV3, with expression mirroring the level of drug resistance associated with each cell line (Fig. 5a). The overexpression of P-gp in SKVLB1 in comparison to parental SKOV3 is dramatic as Fig. 5a required the use of a logarithmic scale to quantify all expression levels. This pattern was also observed with MRP2, albeit at lower expression levels, while MRP1 increased only minimally between the four cell lines. RT-PCR also revealed that neither 0.3 μM cytochalasin A or B influenced the RNA levels of ABC transporters (Fig. 5a). In fact, only 15 μM verapamil influenced RNA expression, with a slight, but noticeable decrease in P-gp levels against all four neoplastic cell lines.

Nevertheless, cytochalasins A and B appeared to have notable inhibitory activity against ABC transporters, as assessed by Rh123 accumulation and efflux analysis (Fig. 5b and c). SKVLB1 cells treated with 0.3 μM cytochalasin A or B readily accumulated Rh123, becoming saturated by the dye at 60 min (Fig. 5b). This activity was also seen with 15 μM verapamil, but not 0.3 μM cytochalasin C, which had only a slight influence on accumulation levels. All treatment groups for SKOV3 became saturated by Rh123, indicative of its low expression of P-gp and other efflux pumps. The activity of cytochalasins A and B, as well as verapamil against ABC transporter-mediated efflux of Rh123 was confirmed by experiments in which cells were incubated with the dye prior to being placed in fresh medium (Fig. 5c). Untreated and 0.3 μM cytochalasin C treated SKVLB1 cells effluxed Rh123 at a consistent rate with untreated cells reaching 12.4 % content of the original Rh123 incubation and cytochalasin C treated cells reaching 17.8 % content. By contrast, 0.3 μM cytochalasins A or B retained much higher percentages of accumulation (62.4 % and 53.1 %, respectively), but were less effective than 15 μM verapamil (68.9 %).

### Efficacy of cytochalasin B in increasing drug sensitivity

Cytochalasin B appeared to increase the drug sensitivity of SKVLB1 cells to clinically approved antineoplastic agents known to have reduced cytotoxicity against this multidrug resistant cell line (Table 4). The IC_90_ values of doxorubicin against SKVLB1 cells at 13.5 h and at 33 h of exposure and for paclitaxel at 13.5 h dropped by 2 to 4-fold using either 0.15 or 0.60 μM cytochalasin B. This increase in sensitivity was even more pronounced with the IC_90_ value of paclitaxel at 33 h, as it dropped 8-fold with 0.15 μM cytochalasin B, and 10-fold with 0.60 μM cytochalasin B. It should be noted that much higher concentrations of doxorubicin and paclitaxel were needed to obtain IC_90_ values against SKVLB1 than in earlier determinations because the earlier values were measured at 96 h of exposure, while the combination of cytochalasin B with doxorubicin or paclitaxel were measured at 13.5 and 33 h.

**Table 4:** Sensitivity to clinically approved antineoplastic agents potentiated by cytochalasin B against multidrug resistant SKVLB1 human ovarian carcinoma

Time of exposure (Hours)	13.5		33	
IC concentrations (μM)	IC_90_	FS	IC_90_	FS
Doxorubicin				
0 μM Cytochalasin B	5	-	5	-
0.15 μM Cytochalasin B	2.5	2	2.5	2
0.60 μM Cytochalasin B	1.3	4	1.3	4
Paclitaxel				
0 μM Cytochalasin B	>40	-	40	-
0.15 μM Cytochalasin B	20	2	5	8
0.60 μM Cytochalasin B	15	2.5	4	10

*FS* fold sensitization

### Assessment of drug synergy between cytochalasins and clinically approved agents

**Fig. 6:**
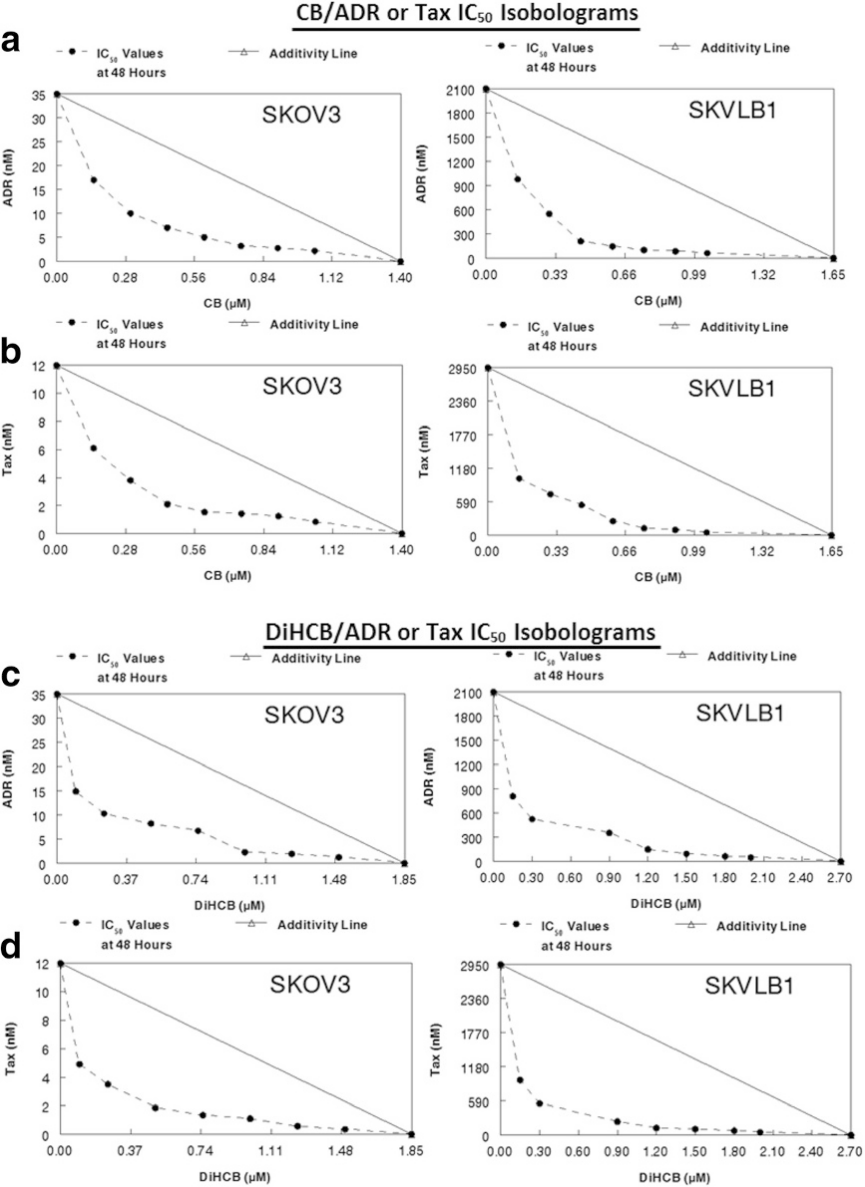
IC_50_ isobolograms for cytochalasin B, 21, 22-dihydrocytochalasin B, doxorubicin, and paclitaxel against SKOV3 and SKVLB1 human ovarian carcinoma. **a** CB and ADR. **b** CB and Tax. **c** DiHCB and ADR. **d** DiHCB and Tax. IC_50_ values were determined after a 48 h continuous exposure

**Fig. 7:**
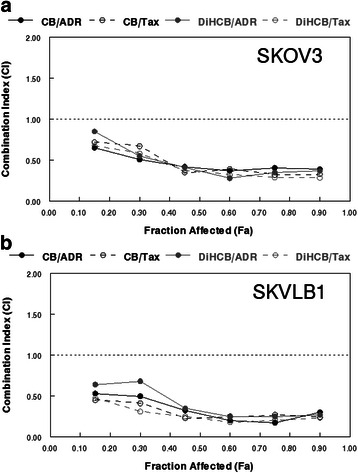
Chou-Talalay method Fa-CI plots to assess drug synergy between cytochalasin B, 21,22-dihydrocytochalasin B, doxorubicin, and paclitaxel against SKOV3 and SKVLB1 human ovarian carcinomas. **a** Concomitant treatments against SKOV3 cells. **b** Concomitant treatments against SKVLB1 cells. Agents were administered continuously for 48 h. According to the Chou-Talalay method for the assessment of drug synergy [27], < 0.1 (very strong synergism), 0.1–0.3 (strong synergism), 0.3–0.7 (synergism), 0.7–0.85 (moderate synergism), 0.85–0.9 (slight synergism) are all values of synergism, while 0.9–1.1 may be considered additive or potentially antagonistic, and any values greater than 1.1 are antagonistic

Both cytochalasin B and DiHCB appeared to synergize with doxorubicin and paclitaxel in SKOV3 and SKVLB1 cells when combined IC_50_ values were plotted to form isobolograms (Fig. 6). The synergy indicated by these values was also confirmed with Chou-Talalay CI values (Table 5). In addition, DRI values for both the cytochalasins and currently approved agents were indicative of favorable dose reductions, although this would be expected from the isobolograms. Interestingly, in both measurements of synergy, it appeared that cytochalasin B and DiHCB synergized with the clinically approved agents more strongly against SKVLB1 than against drug sensitive SKOV3. This synergism was noted at other inhibitory concentrations, as indicated in Fig. 7. Although the individual values may vary, the lines of the Fa-CI plot for SKVLB1 appear to be noticeably lower than those observed for SKOV3. The potential for increased synergy may be the result of the inhibitory effect cytochalasin B and DiHCB has toward P-gp and potentially other ABC transporters, and will be elaborated upon in the discussion.

**Table 5:** Chou-Talalay statistics on the mean IC_50_ values between cytochalasin B, 21, 22-dihydrocytochalasin B, doxorubicin, and paclitaxel against SKOV3 and SKVLB1 human ovarian carcinoma

Cell line	Cytochalasin (μM)	Clinically approved agent (μM)	DRI Cyt^a^	DRI CAA^b^	CI
SKOV3	0.6 CB	0.0067 ADR	3.46	8.20	0.41
	0.6 CB	0.0023 Tax	3.46	6.99	0.43
	0.76 DiHCB	0.0065 ADR	5.23	10.9	0.28
	0.76 DiHCB	0.0030 Tax	5.23	12.5	0.27
SKVLB1	0.6 CB	0.30 ADR	4.07	15.7	0.30
	0.6 CB	0.40 Tax	4.07	19.7	0.29
	1.12 DiHCB	0.29 ADR	5.27	17.5	0.25
	1.12 DiHCB	0.30 Tax	5.27	22.7	0.23

Mean values for drug concentrations, dose reduction indices, and combination indices were derived from the points used in the IC_50_ isobolograms, excluding single agent administrations

*CI* combination index

^a^Dose reduction index for the indicated cytochalasin

^b^Dose reduction index for the indicated currently approved agent

## Discussion

With respect to SK human ovarian carcinoma cells, it appears that the nine cytochalasin congeners examined in this study fall into three groups. Against the SKOV3 cell line, cytochalasins C, D, E and H were less cytotoxic than are the clinically approved antineoplastic agents (doxorubicin, paclitaxel and vinblastine), with 96 h continuous exposure IC_90_ values ranging from 80 to 330 nM (Table 1). IC_90_ values for the three clinically approved agents ranged from 2 to 11 nM. Cytochalasins C, D, E, and H as a group appeared to be subject to drug resistance against multidrug resistant SKVLB1 cells as exemplified by cytochalasin D which had a RI = 100 against SKVLB1. While this RI was not as high as for any of the clinically approved chemotherapeutic agents in the study, it is likely that overexpression of P-gp does reduce the cytotoxicity of these congeners, presumably due to drug efflux.

The second group composed of cytochalasins B, J, DiHCB and DiHCBγ-L were less cytotoxic against the parental cell line SKOV3. IC_90_ values ranged from 0.8 to 40 μM (800 to 40,000 nM), and were not subject to strong resistance by SKVLB1 (RIs ranged from 1 to 4-fold). Further, cytochalasin B, DiHCB, and DiHCBγ-L were more cytotoxic against SKVCR0.015 and SKVCR0.1 than against the parental cell line with RI values below 1 (Table 1).

Finally, Cytochalasin A is unique in that it demonstrated less cytotoxicity against the parental cell line (IC_90_ = 1 μM) than against SKVLB1 (IC_90_ = 0.75 μM), indicating that it is not subject to SKVLB1 drug resistance. This suggests that cytochalasin A has unique properties with respect to inhibiting P-gp, possibly due to increased binding affinity for the drug efflux pump in conjunction with its electrophilic α, β-unsaturated ketone moiety (Fig. 1), which could potentiate alkylation of key structures within the neoplastic cell (including P-gp). The α, β-unsaturated ketone in cytochalasin A is particularly electrophilic, as it is conjugated to another ketone, making the double bond highly reactive.

It is also very likely that DiHCBγ-L is not influenced by SKVLB1 drug resistance, as the IC_90_ values were the same against SKOV3 and SKVLB1. The potential of DiHCBγ-L to inhibit P-gp may be related to its relatively low cytotoxicity. DiHCBγ-L had the highest IC_90_ value against SKOV3 among the nine cytochalasins (40 μM). This suggests that P-gp and other ABC transporters may become saturated by 40 μM DiHCBγ-L in SKVLB1, and that the efflux pumps have difficulty removing such a high concentration. It is also possible that DiHCBγ-L may have increased reactivity deriving from the generation of an α-hydroxyacetamido-group with a reactive secondary hydroxyl group at C-9 when the original lactone of DiHCB is hydrolyzed (Fig. 1), or it may have unique binding capability with ABC transporters. Along with cytochalasin A, DiHCBγ-L may have properties with respect to the drug efflux pump system that would make these two congeners of special interest in preclinical evaluations.

With respect to the intermediately resistant cell line SKVCR0.015, all of the cytochalasins tested (cytochalasins A, B, C, D, DiHCB, and DiHCBγ-L) were equally or more cytotoxic than against SKOV3. All of these cytochalasins except for cytochalasin C also showed equal or enhanced sensitivity rather than resistance when tested against the intermediately resistant cell line SKVCR0.1 (cytochalasins E, H, and J were not tested against the intermediately resistant cell lines). These data suggest that multidrug resistant pumps can be completely overridden by cytochalasins unless there is very high expression of the pumps as in the highly resistant SKVLB1 cell line.

As shown with cytochalasins B, C, D and DiHCB, verapamil was able to increase the cytotoxicity of congeners affected by P-gp overexpression in SKVLB1 cells (Table 3). This increase in cytotoxicity was substantial with the more cytotoxic cytochalasins C and D (32- and 64-fold increase in sensitivity against SKVLB1), which had much higher RIs against SKVLB1 than other congeners. Nevertheless, even less cytotoxic congeners such as cytochalasin B and DiHCB had lower IC_90_ values against all drug resistant cell lines when combined with 30 μM verapamil (Fig. 3a). Further, growth inhibition of SKVCR0.015 cells at varying concentrations of cytochalasin B was more pronounced after the addition of 30 μM verapamil (Fig. 3e), validating previous observations that calcium ion channel blockers can be used to overcome P-gp mediated drug resistance [28–30]. Interestingly, it appeared that verapamil had some propensity to modulate P-gp expression (Fig. 5a), suggesting that it may perturb P-gp function through mechanisms other than direct inhibition of the efflux protein. In addition, verapamil had a slight, but noticeable influence on F/G-actin ratios in both SKOV3 and SKVLB1 (Fig. 4), an observation in accord with prior studies that have noted Ca^2+^ oscillations trigger the depolymerization of microfilaments [31–33]. Together, these data indicate that microfilaments may be a feasible target for inhibiting drug efflux (although the influence verapamil has on microfilaments may merely be an off target effect). This is in accord with Table 3 and Figs. 3 and 4, as concomitant administration of cytochalasin B and verapamil potentiates the cytotoxicity and reduction of F-actin significantly more than cytochalasin B administered as a single agent.

Although verapamil appears to increase the cytotoxicity of most cytochalasins examined in the present study, it is evident that at least some of these congeners have ABC transporter inhibitory activity. As demonstrated in Figs. 5b and c, cytochalasins A and B, but not cytochalasin C, are able to inhibit the ability of SKVLB1 cells to efflux Rh123. These data parallel the 4 day IC_90_ values attained for these agents against SKVLB1, suggesting that the marked cytotoxicity cytochalasin A and potentially other cytochalasins had against the multidrug resistant cell line was at least partially attributed to inhibition of drug efflux. This appears feasible as congeners more cytotoxic against the parental SKOV3 cell line potentiated lower F-actin levels against SKOV3 than they did against SKVLB1, a cell line that has an inherently lower F/G-actin ratio (Fig. 4). By contrast, cytochalasin A elicited a significantly lower F/G-actin ratio against SKVLB1, suggesting that the compound was not being pumped out at as high of a rate as the more potent F-actin inhibitors. In addition, this figure highlights the potential utility of microfilament-disrupting agents, as multidrug resistant SKVLB1 appears to have a lower ratio of F/G-actin than drug sensitive SKOV3, and normal cells often have higher levels of F-actin than their neoplastic counterparts [34, 35]. Disruption of the actin cytoskeleton is known to induce apoptosis in malignant cells [1, 35], and exploiting their already perturbed microfilament network may provide a novel antineoplastic mechanism that warrants clinical investigation.

Besides the potential of using cytochalasin congeners against certain drug resistant cancers, there exists the possibility of concomitant chemotherapy with clinically approved agents to increase their cytotoxicity. As demonstrated in Table 4, sufficient concentrations of cytochalasin B can increase the drug sensitivity of SKVLB1 to doxorubicin and paclitaxel. A formal assessment of drug synergy through isobolographic analysis (Fig. 6) and Chou-Talalay statistics (Fig. 7 and Table 5) further confirmed the potential utility of facilitating the cytotoxicity of currently approved agents with cytochalasins, as both cytochalasin B and DiHCB demonstrated notable synergism with doxorubicin and paclitaxel. The extent of the drug synergy between cytochalasin B, DiHCB, and doxorubicin against SKVLB1 was comparable to the activity we previously observed in P388/ADR leukemia [14], another multidrug resistant cell line. These observations, along with previous studies that noted drug synergy between cytochalasin B and cytarabine [36] as well as vincristine [37], provide compelling evidence that cytochalasin B and its reduced congener have clinically applicable synergistic potential.

These data are in alignment with the antineoplastic mechanisms of microfilament-disrupting agents such as cytochalasin B. Due to cytokinesis inhibition potentiated by this class of agents, neoplastic cells become enlarged and multinucleated; ideal targets for microtubule-directed (paclitaxel) and for nucleic acid-directed (doxorubicin) agents [1, 38]. Further, the concomitant perturbation of the cell cycle via microtubule-targeting G_2_/M arrest and microfilament-targeting cytokinesis inhibition leaves two unique mechanisms that neoplastic cells would have to circumvent in order to continuously proliferate. Indeed, we have previously observed that concomitant administration of cytochalasin B and vincristine substantially precludes the clonogenic potential of U937 human monocytic leukemia, thereby potently inhibiting its propensity to proliferate [39]. Therefore, cytochalasins have unique microfilament-directed mechanisms that may substantiate potent drug synergy with agents that target microtubules or nucleic acids.

Interestingly, we have demonstrated that pulsed low-frequency ultrasound in the 20 to 40 kHz range is able to induce preferential destruction of neoplastic cells enlarged by treatment with cytoskeletal-directed agents [39, 40]. The ability of cytochalasins to affect multidrug resistant neoplastic cells presents the possibility of cell enlargement and multinucleation in these cells that has been observed in all other neoplastic cell lines tested. Consequently, cytochalasins may sensitize multidrug resistant cells to sonodynamic therapy with low frequency ultrasound, paralleling the effects obtained to this point with leukemic cell targets.

## Conclusion

Currently, ovarian carcinoma presents significant clinical issues, as the 5 year survival rate for all stages and types is 44 %, while stage IV invasive ovarian carcinoma has a 5 year survival rate of 17 % [41]. Further, only 15 % of all ovarian tumors are found at stage I [41], indicating that clinicians often have to treat aggressive malignancies. These data suggest that novel antineoplastic agents need to be devised for the treatment of ovarian carcinoma. Cytochalasins offer novel mechanisms for exploitation in cancer therapy that may improve the efficacy of treatments against cancers refractory to standard chemotherapeutic protocols. *In vivo* efficacy of cytochalasins in preclinical models of drug resistant ovarian carcinoma has yet to be determined. Such preclinical investigation is warranted based on the results of this study.

## References

[1] Trendowski M (1846). Exploiting the cytoskeletal filaments of neoplastic cells to potentiate a novel therapeutic approach. Biochim Biophys Acta Reviews on Cancer.

[2] Van Goietsenoven G, Mathieu V, Andolfi A, Cimmino A, Lefranc F, Kiss R (2011). In vitro growth inhibitory effects of cytochalasins and derivatives in cancer cells. Planta Med.

[3] Kelly F, Sambrook J (1973). Differential effect of cytochalasin B on normal and transformed mouse cells. Nat New Biol.

[4] Medina D, Oborn CJ, Asch BB (1980). Distinction between preneopastic and neoplastic mammary cell populations in vitro by cytochalasin B-induced multinucleation. Cancer Res.

[5] Somers KD, Murphey MM (1980). Cytochalasin B-induced multinucleation of human tumor and normal cell cultures. Cell Biol Int Rep.

[6] Steiner MR, Altenburg B, Richards CS, Dudley JP, Medina D, Butel JS (1978). Differential response of cultured mouse mammary cells of varying tumorigenicity to cytochalasin B. Cancer Res.

[7] Somers KD, Murphey MM (1982). Multinucleation in response to cytochalasin B: a common feature in several human tumor cell lines. Cancer Res.

[8] Huang FY, Li YN, Mei WL, Dai HF, Zhou P, Tan GH (2012). Cytochalasin D, a tropical fungal metabolite, inhibits CT26 tumor growth and angiogenesis. Asian Pac J Trop Med.

[9] Małecki JM, Bentke A, Ostrowska B, Laidler P (2010). Cytochalasin D, LY294002 and olomoucine synergize in promoting death of melanoma cells through activation of caspase-3 and apoptosis. Melanoma Res.

[10] Huang FY, Mei WL, Li YN, Tan GH, Dai HF, Guo JL (2012). The antitumour activities induced by pegylated liposomal cytochalasin D in murine models. Eur J Cancer.

[11] Rao JY, Hurst RE, Bales WD, Jones PL, Bass RA, Archer LT (1990). Cellular F-actin levels as a marker for cellular transformation: relationship to cell division and differentiation. Cancer Res.

[12] Ben-Ze'ev A (1985). The cytoskeleton in cancer cells. Biochim Biophys Acta..

[13] Bousquet PF, Paulsen LA, Fondy C, Lipski KM, Loucy KJ, Fondy TP (1990). Effects of cytochalasin B in culture and in vivo on murine Madison 109 lung carcinoma and on B16 melanoma. Cancer Res.

[14] Trendowski M, Mitchell JM, Corsette CM, Acquafondata C, Fondy TP (2015). Chemotherapy with cytochalasin congeners in vitro and in vivo against murine models. Invest New Drugs.

[15] Smith CD, Carmeli S, Moore RE, Patterson GM (1993). Scytophycins, novel microfilament-depolymerizing agents which circumvent P-glycoprotein-mediated multidrug resistance. Cancer Res.

[16] Shaw TJ, Senterman MK, Dawson K, Crane CA, Vanderhyden BC (2004). Characterization of intraperitoneal, orthotopic, and metastatic xenograft models of human ovarian cancer. Mol Ther.

[17] Anglesio MS, Wiegand KC, Melnyk N, Chow C, Salamanca C, Prentice LM (2013). Type-specific cell line models for type-specific ovarian cancer research. PLoS One.

[18] Bradley G, Naik M, Ling V (1989). P-glycoprotein expression in multidrug-resistant human ovarian carcinoma cell lines. Cancer Res.

[19] Speicher LA, Barone LR, Chapman AE, Hudes GR, Laing N, Smith CD (1994). P-glycoprotein binding and modulation of the multidrug-resistant phenotype by estramustine. J Natl Cancer Inst.

[20] Chabner BA, Longo DL. Cancer chemotherapy and biotherapy: principles and practice, 5th Edition. Philadelphia, PA: Lipincott Williams & Wilkins; 2011.

[21] Yusa K, Tsuruo T (1989). Reversal mechanism of multidrug resistance by verapamil: direct binding of verapamil to P-glycoprotein on specific sites and transport of verapamil outward across the plasma membrane of K562/ADM cells. Cancer Res.

[22] Futscher BW, Foley NE, Gleason-Guzman MC, Meltzer PS, Sullivan DM, Dalton WS (1996). Verapamil suppresses the emergence of P-glycoprotein-mediated multi-drug resistance. Int J Cancer.

[23] Fojo AT, Shen DW, Mickley LA, Pastan I, Gottesman MM (1987). Intrinsic drug resistance in human kidney cancer is associated with expression of a human multidrug-resistance gene. J Clin Oncol.

[24] Mickisch GH, Merlino GT, Galski H, Gottesman MM, Pastan I (1991). Transgenic mice that express the human multidrug-resistance gene in bone marrow enable a rapid identification of agents that reverse drug resistance. Proc Natl Acad Sci USA.

[25] Lipski KM, McQuiggan JD, Loucy KJ, Fondy TP (1987). Cytochalasin B: preparation, analysis in tissue extracts, and pharmacokinetics after intraperitoneal bolus administration in mice. Anal Biochem.

[26] Trendowski M, Wong V, Wellington K, Hatfield S, Fondy TP (2014). Tolerated doses in zebrafish of cytochalasins and jasplakinolide for comparison with tolerated doses in mice in the evaluation of pre-clinical activity of microfilament-directed agents in tumor model systems in vivo. In Vivo.

[27] Chou TC (2006). Theoretical basis, experimental design, and computerized simulation of synergism and antagonism in drug combination studies. Pharmacol Rev.

[28] Presant CA, Kennedy PS, Wiseman C, Gala K, Bouzaglou A, Wyres M (1986). Verapamil reversal of clinical doxorubicin resistance in human cancer. A Wilshire Oncology Medical Group pilot phase I-II study. Am J Clin Oncol.

[29] Miller TP, Grogan TM, Dalton WS, Spier CM, Scheper RJ, Salmon SE (1991). P-glycoprotein expression in malignant lymphoma and reversal of clinical drug resistance with chemotherapy plus high-dose verapamil. J Clin Oncol.

[30] Taylor CW, Dalton WS, Mosley K, Dorr RT, Salmon SE (1997). Combination chemotherapy with cyclophosphamide, vincristine, adriamycin, and dexamethasone (CVAD) plus oral quinine and verapamil in patients with advanced breast cancer. Breast Cancer Res Treat.

[31] Rosenmund C, Westbrook GL (1993). Calcium-induced actin depolymerization reduces NMDA channel activity. Neuron.

[32] Dartsch PC, Ritter M, Häussinger D, Lang F (1994). Cytoskeletal reorganization in NIH 3 T3 fibroblasts expressing the ras oncogene. Eur J Cell Biol.

[33] Lang F, Busch GL, Ritter M, Völkl H, Waldegger S, Gulbins E, Häussinger D (1998). Functional significance of cell volume regulatory mechanisms. Physiol Rev.

[34] Stournaras C, Stiakaki E, Koukouritaki SB, Theodoropoulos PA, Kalmanti M, Fostinis Y, Gravanis A (1996). Altered actin polymerization dynamics in various malignant cell types: evidence for differential sensitivity to cytochalasin B. Biochem Pharmacol.

[35] Desouza M, Gunning PW, Stehn JR (2012). The actin cytoskeleton as a sensor and mediator of apoptosis. Bioarchitecture.

[36] O'Neill FJ (1975). Selective destruction of cultured tumor cells with uncontrolled nuclear division by cytochalasin B and cytosine arabinoside. Cancer Res.

[37] Kolber MA, Hill P (1992). Vincristine potentiates cytochalasin B-induced DNA fragmentation in vitro. Cancer Chemother Pharmacol.

[38] Trendowski M (2015). Using cytochalasins to improve current chemotherapeutic approaches. Anticancer Agents Med Chem.

[39] Trendowski M, Wong V, Zoino JN, Christen TD, Gadeberg L, Sansky M, Fondy TP (2015). Preferential enlargement of leukemia cells using cytoskeletal-directed agents and cell cycle growth control parameters to induce sensitivity to low frequency ultrasound. Cancer Lett.

[40] Trendowski M, Yu G, Wong V, Acquafondata C, Christen T, Fondy TP (2014). The real deal: using cytochalasin B in sonodynamic therapy to preferentially damage leukemia cells. Anticancer Res.

[41] Siegel RL, Miller KD, Jemal A (2015). Cancer statistics, 2015. CA Cancer J Clin.

